# Montreal Cognitive Assessment Is Superior to Standardized Mini-Mental Status Exam in Detecting Mild Cognitive Impairment in the Middle-Aged and Elderly Patients with Type 2 Diabetes Mellitus

**DOI:** 10.1155/2013/186106

**Published:** 2013-07-11

**Authors:** Kannayiram Alagiakrishnan, Nancy Zhao, Laurie Mereu, Peter Senior, Ambikaipakan Senthilselvan

**Affiliations:** ^1^Division of Geriatric Medicine, Department of Medicine, University of Alberta, Edmonton, AB, Canada T6G 2G3; ^2^University of Alberta Hospital, B139 Clinical Sciences Building, 8440-112 Street, Edmonton, AB, Canada T6G 2G3; ^3^Department of Medicine, University of Alberta, Edmonton, AB, Canada T6G 2G3; ^4^Division of Endocrinology, Department of Medicine, University of Alberta, Edmonton, AB, Canada T6G 2G3; ^5^School of Public Health, University of Alberta, Edmonton, AB, Canada T6G 2G3

## Abstract

*Aim*. This study compares the usefulness of Montreal Cognitive Assessment (MoCA) to Standardized Mini-Mental Status Exam (SMMSE) for diagnosing mild cognitive impairment (MCI) in Type 2 diabetes mellitus (DM) population. *Methods*. This prospective pilot study enrolled 30 community dwelling adults with Type 2 DM aged 50 years and above. Subjects were assessed using both the SMMSE and MoCA for MCI. In all subjects, depression and dementia were ruled out using the DSM IV criteria, and a functional assessment was done. MCI was diagnosed using the standard test, the European consortium criteria. Sensitivity and specificity analysis, positive and negative predictive values, likelihood ratios and Kappa statistic were calculated. *Results*. In comparison to consortium criteria, the sensitivity and specificity of MoCA were 67% and 93% in identifying individuals with MCI, and SMMSE were 13% and 93%, respectively. The positive and negative predictive values for MoCA were 84% and 56%, and for SMMSE were 66% and 51%, respectively. Kappa statistics showed moderate agreement between MoCA and consortium criteria (kappa = 0.4) and a low agreement between SMMSE and consortium criteria (kappa = 0.07). *Conclusion*. In this pilot study, MoCA appears to be a better screening tool than SMMSE for MCI in the diabetic population.

## 1. Background

Diabetes Mellitus (DM) is a common metabolic disorder worldwide and is seen in 11.3% of people in the age group of 20 or older and 26.9% in 65 years and older in the United States [1]. The prevalence and incidence of DM increase with aging. DM is associated with increased risk for mild cognitive impairment (MCI) in the middle-aged and elderly population [2, 3], but also seems to accelerate the progression of MCI to dementia in elderly people with DM [4]. A meta-analysis of longitudinal studies showed diabetes increased the risk of mild cognitive impairment by 21% [5]. Diabetes was also related to a significantly higher risk for all-cause MCI, amnestic (memory domain) and nonamnestic (nonmemory domain) MCI [6].

Mini-Mental Status examination (MMSE) has been used as a global screening tool for cognitive impairment for the last three decades, but in clinical practice it is not sensitive in detecting mild cognitive impairment. Many other assessment scales have been developed to assess cognitive impairment. Among these, the Montreal Cognitive Assessment (MoCA) was developed to screen for MCI [7], but thus far MoCA has not been validated in people with DM. The aim of this study was to compare the usefulness of MoCA with Standardized Mini-Mental Status Exam (SMMSE) in diagnosing MCI in middle-aged and elderly subjects with type 2 DM. 

## 2. Methodology

This was a prospective observational pilot study, in 30 consecutive diabetic subjects of both genders and age greater than 50 years, who attended the diabetes education clinics in Edmonton, Alberta, Canada. Patients with a history of dementia, blindness, stroke, and known depression were excluded from the study. MoCA [7] and SMMSE [8] were performed in all patients to screen for cognitive impairment. The different cognitive domains assessed in these tests are shown in Table 1.

The SMMSE has timed tasks and strict guidelines for administration. As a result, this test has lower inter- and intrarater variability when compared to MMSE. The SMMSE measures 6 cognitive domains, and in this study the scores were also corrected for age and education [9]. Traditionally, a MMSE cut-off score of 24 or less is significant, but with SMMSE after correcting for age and education, the cut off varies from 19 to 29 based on different age groups from >18 to >84, as well as education from fourth grade to college education.

MoCA measures 7 cognitive domains and includes domains which are not measured by SMMSE like executive function and abstraction. MoCA has excellent test-retest reliability, and the internal consistency on the items in MoCA was 0.83 [7]. A MoCA score of 26 or less is considered as MCI. Since depression can cause cognitive deficits, it was ruled out by using DSM IV criteria for depression [10]. Dementia was also ruled out using DSM IV criteria for dementia [10]. Functional information on daily activities was collected using Katz Basic Activities of Daily living (BADL) and Lawton Instrumental Activities of Daily Living (IADL) questionnaires [11, 12]. BADL like bathing, dressing, toileting, transferring, continence, and feeding as well as IADL like using telephone, housekeeping, laundry, shopping, food preparation, transportation, medication, and finance management was also assessed. Initial criteria for MCI were proposed by Petersen [13]. Modification of Petersen's criteria as proposed by the European Consortium on Alzheimer's disease was used as the currently available standard test to diagnose MCI [13, 14]. The criteria include (1) cognitive complaints coming from the patients or their families, (2) the reporting of a decline in cognitive functioning relative to previous abilities during the past year by the patient or informant, (3) cognitive disorders as evidenced by clinical evaluation (impairment in memory or in another cognitive domain, which in this study was assessed by SMMSE and MoCA), (4) absence of major repercussions on daily life (in this study, measured by Katz ADL and Lawton IADL), and (5) absence of dementia (in this study, dementia was ruled out by using DSM IV criteria). Ethics approval was obtained from the University of Alberta ethics board.

## 3. Statistical Analysis

Descriptive statistics were performed on all demographic and clinical parameters. The differences in the means of continuous variables were tested by *t*-tests. The differences in the proportion were tested by chi-square or Fisher's exact tests. MoCA and SMMSE assessed a range of cognitive skills on a scale of 0–30 points. The cutoffs for suggested mild cognitive impairment used in this study were scores between 19 and 29, (scores corrected based on age and education) for SMMSE, and scores ≤26 for MoCA with a one-point adjustment to the total score for subjects with less than 12 years of education. Diagnosis of MCI using European consortium criteria (Yes or No) was compared with the dichotomized SMMSE (normal or abnormal) and dichotomized MoCA (normal or abnormal), respectively. Sensitivity and specificity analysis, positive and negative predictive values, and likelihood ratios were done. Using the receiver operating characteristic analysis (ROC), the discriminatory ability of MoCA and SMMSE to determine cognitive impairment was examined. The kappa statistic was used to assess agreement.

## 4. Results

Mean age of the study subjects was 59.9 years (SD: 7.1). Twenty-two (73%) of the study subjects were middle aged (50–64 years) and 14 (47%) of the study subjects were females. The average duration of diabetes was 4.5 years (SD: 5.9) with 12 (40%) treated with insulin. Mean duration of DM was less in the MCI group compared with non-MCI group. Using the standard European consortium criteria, the prevalence of MCI was 50% (15/30) in the whole group and 36% (8/22) in middle-aged subjects. Amnestic MCI was seen in 13 out of 15 subjects and two had nonamnestic MCI in this study. The baseline characteristics of the subjects with and without MCI by European consortium criteria were shown in Table 2. 

**Figure 1:**
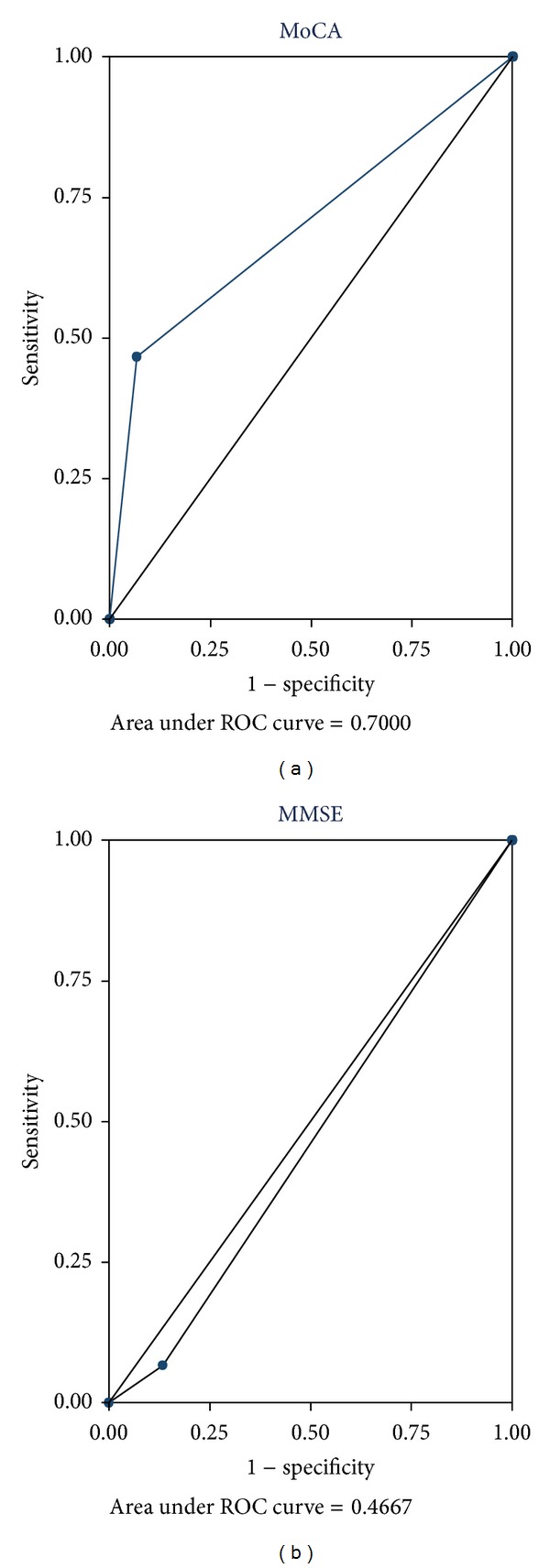
ROC curves for the comparison of agreement between MoCA and SMMSE.

After correcting for age and education, three subjects (10%) had abnormal SMMSE scores, whereas 8 (27%) had MoCA scores less than 26 suggesting mild cognitive impairment. In comparison to the European consortium criteria, the sensitivity and specificity of MoCA were 67% and 93% and of SMMSE were 13% and 93%, respectively. The positive and negative predictive values for MoCA were 84% and 56% and for SMMSE were 66% and 51%, respectively. Positive likelihood ratio for MoCA was 9.5 and for SMMSE was 1.8. Agreement was moderate between MoCA and European consortium criteria (kappa = 0.4) but low between SMMSE and European consortium criteria (kappa = 0.07). As shown in Figure 1, the discriminatory ability for MoCA to diagnose MCI as represented by an area under the ROC curve was fair (0.70) but superior to that of SMMSE (0.47). Specific difficulties in cognitive domains like abstraction, executive function (clock drawing), visuospatial function and delayed 5-word recall in MoCA appear to help this test as a better screening procedure for MCI (Tables 3 and 4).

## 5. Discussion

 In this study, MoCA has a better sensitivity than SMMSE in diagnosing MCI and has a moderate agreement with modified Petersen's/European consortium criteria. In this study, SMMSE was not sensitive for identifying early cognitive changes associated with MCI, which was also shown in another study by Tang-Wai et al. [15]. To our knowledge, this is the first study that has compared the usefulness of MoCA with SMMSE in screening for MCI, in middle-aged and elderly people with type 2 DM. Since MoCA assesses a broader range of cognitive domains including abstraction and executive function, it may be more sensitive than SMMSE to diagnose MCI in DM subjects. 

Prevalence and incidence information about MCI in diabetes is sparse in the literature. In a small case control study of type 2 DM, prevalence of CIND (Cognitive Impairment No Dementia) was 38% compared to 20% in the controls [16]. A Japanese study showed 29% of the study subjects whose MMSE score ranges between 24 and 27 had a diagnosis of MCI [17]. A population-based study reported that the incidence of MCI in diabetic subjects was around 28% [6]. Diabetes might also accelerate the conversion of MCI to dementia (HR: 2.9, 95% CI 1.1–7.3) [18]. In this study MCI was seen in a good proportion, 8/22 (36%) of middle-aged subjects, when compared to older age DM subjects. Study results indicate MCI was seen with less duration of DM when compared to the non-MCI group. It is possible that other factors like severity of the disease and hypoglycemic incidents may be contributing to MCI, but this study does not have information about it.

 This study points out by using a better scale like MoCA may help to identify cognitive impairment in patients with diabetes, which is associated with long term risk of cognitive decline and later dementia in a sizeable fraction of patients. The MoCA is recognized as superior to the SMMSE for detecting mild stages of cognitive impairment, as it requires a broader range of cognitive processes for perfect scores, and all items are explicitly related to key domains of cognitive impairment. Simple cognitive test like MoCA is likely to be useful when screening in large community samples, where detailed clinical histories and assessment may not be available to fulfill the European consortium criteria for MCI. For this population, quality normative data are also scarce. The understanding of early or subtle cognitive changes in diabetes and the identification of a group who are at risk for developing dementia are important from a preventive perspective. Since cognitive impairment may result in poor adherence with home blood glucose monitoring, dietary and medication management, and followup with the healthcare team, screening using a valid cognitive scale should be done. Limitations includes the following: this study had only volunteered community diabetic subjects, with no control group. Because of the small sample size, the generalizability of these study results is limited. 

## 6. Conclusion

MoCA appears to be a better screening test than SMMSE for detecting MCI in middle-aged and elderly patients with type 2 DM. The traditional method to diagnose cognitive impairment using SMMSE may likely need to be revised with MoCA to effectively identify affected diabetic subjects in the community setting. Future, larger prospective studies should be done to verify the findings in this study and also to assess the ideal screening tool in detecting MCI.

### 6.1. Novelty Statement/Important Findings

- Mild cognitive impairment (MCI) was seen even in middle-aged patients with type 2 DM in the community.
- Study results indicate MCI was seen with less duration of DM, when compared to the non-MCI group. It is possible that other factors like severity of the disease and hypoglycemic incidents may be contributing to MCI, but this study did not have information about it.
- MoCA appears to be a better screening test than MMSE for diagnosing MCI in this population however large prospective study is needed to confirm this finding.

## Figures and Tables

**Table 1:** Cognition domains tested in SMMSE and MoCA.

SMMSE	MoCA
Orientation	Orientation
Registration	Abstraction
Attention and calculation	Attention and calculation
Recall	Recall
Language	Language
Visuospatial function	Visuospatial function
	Executive function

**Table 2:** Baseline characteristics by MCI status based on the European consortium criteria*.

Baseline characteristics	MCI present (*n* = 15)	MCI absent (*n* = 15)	*P* value
Age (mean/SD)	60.7 (7.9)	59.1 (6.2)	0.52
Male sex	9/15 (60.0%)	7/15 (47.7%)	0.46
Duration of diabetes (in years/SD)	3.3 (4.2)	5.8 (7.1)	0.24
Insulin	4/15 (26.7%)	8/15 (53.3%)	0.14
Oral agents	11/15 (77%)	14/15 (93%)	0.12
Education (mean/SD)	11.1 (1.2)	11.2 (1.4)	0.79
SMMSE (mean/SD)	29.4 (0.7)	29.3 (1.4)	0.87
MoCA (mean/SD)	25.6 (2.2)	27.3 (3.0)	0.09
DSM IV-depression score (mean/SD)	1.60 (1.18)	0.86 (1.25)	0.11
Functional decline (ADL/IADL)	0/15 (0%)	1/15 (7%)	1.0*

*The differences in the means of continuous variables were tested by *t*-tests. With discrete variable, the differences in the proportions were tested by chi-square tests except that Fisher's exact test was used to test the differences in the functional decline.

**Table 3:** Abnormal scores on different domains of MoCA in both middle-aged (50–64 years) and elderly (≥65 years) patients with and without MCI by the European consortium criteria.

Abnormal score on domains	European consortium criteria		*P* value
	MCI present (*n* = 15)	MCI absent (*n* = 15)	
Total score (≤26)	7 (46.7%)	1 (6.7%)	0.04
Visuo spatial	8 (53.3%)	6 (40%)	0.46
Language	8 (53.3%)	4 (26.7%)	0.14
Attention and calculation	6 (40%)	2 (13.3%)	0.22*
Abstraction	6 (40%)	3 (20%)	0.43*
Memory	13 (86.7%)	10 (66.1%)	0.39*
Orientation	0 (0%)	0 (0%)	—
Clock drawing	6 (40%)	2 (13.3%)	0.22

With discrete variable, the differences in the proportions were tested by chi-square tests except that *Fisher's exact test was used to test the differences in the attention and calculation, abstraction, and memory.

**Table 4:** Abnormal scores on different domains of MoCA in the middle-aged (50–64 years) patients with and without MCI by the European consortium criteria.

Abnormal score on MoCA	European consortium criteria		*P* value*
	MCI present (*n* = 11)	MCI absent (*n* = 11)	
Total score (≤26)	4 (36.4%)	1 (9.1%)	0.31
Visuo spatial	4 (36.4%)	3 (27.3%)	1.00
Language	5 (45.5%)	6 (54.6%)	1.00
Attention and calculation	4 (36.4%)	1 (9.1%)	0.31
Abstraction	6 (54.6%)	3 (27.3%)	0.39
Memory	10 (90.9%)	7 (63.6%)	0.31
Orientation	0 (0.0%)	0 (0.0%)	—
Clock drawing	3 (27.2%)	2 (18.2%)	1.0

*The differences in the proportions were tested by Fisher's exact test.
